# Elderly outpatient profile and predictors of falls

**DOI:** 10.1590/S1516-31802013000100003

**Published:** 2013-02-01

**Authors:** Grace Angélica de Oliveira Gomes, Fernanda Aparecida Cintra, Fernanda Sotelo Batista, Anita Liberalesso Neri, Maria Elena Guariento, Maria da Luz Rosario de Sousa, Maria José D’Elboux

**Affiliations:** I MSc. Physical Educator, Bioscience Institute, Universidade Estadual Paulista (Unesp), Rio Claro, São Paulo; and Department of Gerontology, Universidade Federal de São Carlos (UFSCar), São Carlos, São Paulo, Brazil.; II PhD. Professor, Faculty of Nursing, Universidade Estadual de Campinas (Unicamp), Campinas, São Paulo, Brazil.; III MSc. Physiotherapist, Municipal Health Department of Santos, Santos, São Paulo, Brazil.; IV PhD. Full Professor, Department of Education, Universidade Estadual de Campinas (Unicamp), Campinas, São Paulo, Brazil.; V PhD. Professor, Department of Internal Medicine, Faculdade de Ciências Médicas, Universidade Estadual de Campinas (Unicamp), Campinas, São Paulo, Brazil.; VI PhD. Professor, Department of Dentristy, Faculdade de Odontologia de Piracicaba (FOP), Universidade Estadual de Campinas (Unicamp), Piracicaba, São Paulo, Brazil.

**Keywords:** Activities of daily living, Postural balance, Gait, Muscle strength, Aging, Atividades cotidianas, Equilíbrio postural, Marcha, Força muscular, Envelhecimento

## Abstract

**CONTEXT AND OBJECTIVES:**

Falls are a serious public health problem and are one of the biggest reasons for hospitalization, morbidity and mortality among elderly people. Moreover, few studies on predictors of falls have been conducted in low and middle income countries. The aim here was to identify elderly outpatient profiles according to sociodemographic, clinical, physical and functional variables and correlate them with occurrences of falls among these subjects.

**DESIGN AND SETTING:**

Cross-sectional descriptive study forming part of the project “Quality of Life of Frail Elderly People”, carried out in Campinas, Brazil.

**METHODS:**

The subjects were 145 elderly individuals (76.3 ± 7.8 years old), of whom 65% were women, who were living in the city of Campinas or nearby and were attended at the geriatric outpatient clinic of a University Hospital. Sociodemographic, clinical, physical and functional data, as well as fall occurrence data, were gathered. Cluster analyses and comparisons between groups were carried out.

**RESULTS:**

Cluster analysis identified two distinct groups related to the study variables, and the determinants for this distinction were: gender, marital status, physical performance, handgrip strength and functional independence. These groups were compared according to occurrences of falls over the last year, and significant differences between them were found.

**CONCLUSIONS:**

The results showed that greater occurrences of falls were associated with a profile of elderly people comprising female gender, single status, lower muscle strength and physical performance regarding balance and gait, and lower independence in motor tasks for activities of daily living.

## RESUMO

**CONTEXTO E OBJETIVO:**

Quedas são um sério problema de saúde pública e um dos maiores responsáveis por hospitalização, morbidade e mortalidade em idosos. Além disso, existem poucos estudos sobre preditores de quedas em países de renda baixa e média. O objetivo foi identificar perfis de idosos em acompanhamento ambulatorial segundo variáveis sociodemográficas, clínicas, físicas e funcionais e relacioná-los à ocorrência de quedas desses sujeitos.

**TIPO DE ESTUDO E LOCAL:**

Estudo transversal e descritivo, parte do Projeto “Qualidade de Vida de Idosos Frágeis”, realizado em Campinas, Brasil.

**MÉTODOS:**

Os sujeitos eram 145 idosos (76,3 ± 7,8 anos), sendo 65% mulheres, residentes na região de Campinas, São Paulo, atendidos no ambulatório de geriatria de um hospital universitário de Campinas. Os dados coletados foram: sociodemográficos, clínicos, físicos, funcionais e ocorrência de quedas. Foram realizadas as análises de conglomerados (*cluster analysis*) e de comparação entre os grupos.

**RESULTADOS:**

A análise de conglomerados identificou dois grupos distintos em relação às variáveis do estudo, sendo as determinantes para esta distinção as seguintes: gênero, estado civil, desempenho físico, força de preensão manual e independência funcional. Esses grupos foram comparados segundo a ocorrência de quedas no último ano e identificaram-se diferenças significativas entre eles.

**CONCLUSÕES:**

Os resultados apontam que a maior ocorrência de quedas relacionou-se com o perfil de idosos do sexo feminino, solteiros (as), com piores níveis força muscular, desempenho físico de equilíbrio e marcha e, ainda, menor independência em tarefas motoras das atividades da vida diária.

## INTRODUCTION

Falls are frequent phenomena in individuals over 65 years of age and they have the capability to cause functional decline and weakening of health, thus increasing elderly people’s frailness and vulnerability to morbidity and mortality.^1^^,^^2^^,^^3^ In some cases, the functional incapacity caused by the falls can lead them to dependence on other people to carry out daily activities for them, for long periods or even until death.^4^ For this reason and others associated with hospitalization, treatment and rehabilitation costs, falls among elderly people are recognized as a public health issue.^5^

Elderly people’s level of mobility is one of the essential standards in accomplishing tasks that define functional independence. The ability to perform daily activities can become modified through declining mobility and physical ability, which relates to reduction in the levels of muscle strength, difficulty in executing gait and alterations to static balance.^6^ Occurrences of falls at advanced age not only bring social consequences but also affect economic, physical and psychological factors.^7^ Elderly people frequently seek outpatient treatment for these complications, which interfere with their functionality and quality of life.

Perracini and Ramos^7^ considered that it was important to undertake clinical evaluations and scientific research with the aims of identifying the profile of elderly people who are prone to falls and those who are more vulnerable to serious injuries caused by this type of accident. However, in Brazil, there is a lack of preventive actions at primary and secondary care levels regarding falls among elderly people, and few population-based studies have been conducted on the factors associated with such phenomena. Among the studies available, the ones by Perracini and Ramos^7^ and Siqueira et al.^3^ are of great importance.

Elderly people attending geriatric outpatient clinics constitute a particular healthcare group whose characteristics are very specific and depend on the type of institution that they attend. These individuals present characteristics differing from those of the ordinary population and, therefore, deeper evaluation taking into account the possibility of constructing and applying preventive clinic strategies is needed. Thus, for this population, there is a need to ascertain its profile with regard to falls, and to determine whether this is similar to what is observed in other institutions and communities. Consequently, it is desirable to establish the profile of falls among the geriatric outpatient clinical population, in order to analyze the differences in relation to other institutions and communities and to establish care strategies for older adults.

## OBJECTIVE

The present study aimed to identify elderly outpatient profiles according to sociodemographic, clinical, physical and functional variables and correlate these with occurrences of falls.

## METHODS

This cross-sectional study was carried out in Campinas, São Paulo, Brazil. The population evaluated was composed of 145 subjects over 60 years of age, of both sexes, living in the city of Campinas or nearby. They were attended at the Geriatric Outpatient Clinic of the hospital of Campinas State University (Universidade Estadual de Campinas, Unicamp) between 2005 and 2007. These subjects agreed to participate in a project called “Quality of Life of Frail Elderly People”, which was developed at this clinic after approval by the institution’s Research Ethics Committee. The patients were given assurances regarding anonymity and their freedom to withdraw from the study at any time, when they signed a consent statement.

Data were gathered through individual interviews, with or without the presence of the caregiver, immediately before the initial consultation. A non-probabilistic convenience sample and the following inclusion criteria were used: acceptance of participation in the research; existence of conditions for establishing oral communication, for the interview to be conducted; and Mini-Mental State Examination score higher than the threshold level, as defined by Bertolucci et al.^8^ The following exclusion criteria were taken: refusal to participate; impaired oral communication; presence of a cognitive deficit that made comprehension difficult; and Mini-Mental State Examination score lower than the reported elimination grade.

As many elderly people as possible were approached each day, and they entered the study if they agreed to participate, had time available for the interview and fulfilled the inclusion criteria. In this manner, we were able to interview two elderly people a day.

The following tools were used to gather data: sociodemographic characterization, in which information on gender, age and marital status was obtained; clinical characterization, in which occurrences of falls over the last year (number and resultant fractures and hospitalizations), number of reported diseases, number of medications and visual acuity (using the Snellen optometric chart) were ascertained; characterization of the level of physical activity, which included physical performance measured by means of the Short Physical Performance Battery (SPPB) in the following domains: balance, gait, lower-limb strength,^9^ handgrip strength through Crown dynamometer and functional capacity through the Functional Independence Measure (FIM).^10^^,^^11^^,^^12^

The statistical analysis included cluster analysis and the chi-square test for the categorical data. In view of the fall predictors used in the literature and our outcome, we used cluster analysis because it is able to consistently identify groups and their characteristics. In addition, the Kolmogorov-Smirnov test was used, as well as Student’s t test and the Mann-Whitney test, which were applied to the scale data. The significance level adopted was 5% (P-value < 0.05).

## RESULTS

Most of the individuals were women (65.0%) and aged 75 or over (53.1%). A significant number had suffered falls during the preceding year (51.0%). Among these, 56.2% had fallen at least twice; 64.5% presented poor vision; 18.0% suffered fractures caused by the fall and 19.0% were admitted to hospital because of the fall. The individuals presented an average of 5.3 illnesses (± 2.3) and were taking an average of 5.1 medications (± 2.3) (Table 1).

**Table 1.** Description of 145 elderly subjects’ sociodemographic, clinical, physical and functional variables

Variables	n (%)	Mean (? SD^b^)	Observed variation	Possible range
Gender				
Male	52 (35.0%)			
Female	93 (65.0%)			
Age				
60-74 years	67 (45.6%)	76.3 (? 7.8)	60-93	
³ 75 years	78 (53.1%)			
Marital status				
Married	93 (64.1%)			
Not married	52 (35.8%)			
Falls				
Yes	74 (51.0%)			
No	71 (49.0%)			
Number of falls over the last year				
1	32 (43.8%)	2.2 (? 1.9)	1-15	
³ 2	40 (56.2%)			
Number of illnesses		5.3 (? 2.3)	1-13	
Number of medications		5.1 (? 2.3)	1-13	
Poor vision^*^				
Yes	91 (64.5%)			
No	50 (35.5%)			
Physical activity				
Yes	41 (28.2%)			
No	104 (71.8%)			
Physical performance (SPPB)				
Balance		2.7 (? 1.3)	0-4	0-4
LL strengthª		1.1 (? 1.0)	0-4	0-4
Gait		1.1 (? 0.8)	0-3	0-4
Total		5.9 (? 2.6)	0-10	0-12
Handgrip strength (kg)		20.6 (? 8.1)	7.6-49.6	0-50
Functional independence				
Motor		82.1 (? 9.6)	42-91	13-91
Measure (FIM)				
Total		112.8 (? 13.1)	62-126	18-126

ªLL = lower limb; bSD = standard deviation;*Poor vision = visual acuity less than 20/60; FIM = Functional Independence Measure.

Only 28.2% of the elderly people practiced regular physical activity. They presented low physical performance, as assessed by SPPB, with a total value of 5.9 (± 2.6). They had high scores for functional independence measured by FIM, with a total mean score of 112.8 (± 13.1).

Cluster analysis identified two distinct groups in relation to the study variables:

- Group 1: mostly composed by married men, aged 75 or over, with high numbers of illnesses and medications in use, high prevalence of poor vision, less sedentary, with better levels of handgrip strength (HS), physical performance and functional independence.
- Group 2: mostly composed by single elderly women, aged 75 or over, with high numbers of illnesses and medications in use, low prevalence of poor vision, more sedentary, with worse levels of handgrip strength (HS), physical performance and functional independence.

The groups were compared considering the variables that were determinant for characterization of the groups, and significant differences were found concerning gender, marital status, handgrip strength, physical performance (especially balance), gait, total SPPB score and functional independence in the motor dimension of FIM, for the following tasks: having a bath, dressing (upper body), bladder control, toilet, transfer, use of stairs and total FIM score (Tables 2 and 3).

**Table 2.** Comparison between the clusters according to sociodemographic and clinical variables (n = 145)

Variables	Group 1 (n = 46)		Group 2 (n = 94)		P-value^*^
	n	%	n	%	
Gender					
Male	39	47.8%	13	44.6%	< 0.001
Female	7	52.2%	81	55.4%	
Marital status					
Yes	30	65.2%	31	44.6%	< 0.001
No	16	34.7%	63	55.4%	
Age group					
60-74 years	22	47.8%	42	44.6%	0.173
³ 75 years	24	52.2%	52	55.4%	
Poor vision					
No	21	45.6%	56	58.6%	0.174
Yes	12	54.3%	35	41.3%	
Associated illnesses					
0-4	18	39.1%	31	32.9%	0.474
³ 5	28	60.9%	63	67.1%	
Number of medications					
0-4	21	45.6%	27	28.7%	0.083
³ 5	25	54.3%	64	71.2%	

*Chi-square test; P-value < 0.05.

**Table 3.** Comparison of the clusters according to physical and functional variables (n = 145)

Variables	Group 1 (n = 46)		Group 2 (n = 94)		P-value
	n (%)	Mean (± SD^a^)	n (%)	Mean (± SD^a^)	
Regular physical activity					
Yes	17 (36.9)		24 (25.5)		0.933^*^
No	29 (63.1)		70 (74.4)		
Handgrip strength^1^		30.6 (± 6.0)		16.0 (± 3.6)	P < 0.001^†^
Physical performance (SPPB)					
Balance^2^		3.2 (± 1,1)		2.5 (± 1.3)	0.006^‡^
LL strength^2 a^		1.3 (± 1.0)		1.0 (± 0.7)	0.840^‡^
Gait^2^		2.4 (± 1.0)		1.8 (± 1.0)	0.010^‡^
Total SPPB^3 b^		7.3 (± 2.1)		5.5 (± 2.6)	0.010^†^
Functional performance					
*FIM items* ^*c*^					
Feeding^4^		6.8 (± 0.5)		6.7 (± 0.8)	0.607^†^
Motor self-care					
Grooming		6.9 (± 0.3)		6.8 (± 0.7)	0.297^†^
Having a bath		6.7 (± 0.6)		6.3 (± 1.3)	0.013^†^
Dressing upper body		6.9 (± 0.3)		6.5 (± 1.1)	0.015^†^
Dressing lower body		6.5 (± 0.7)		6.1 (± 1.2)	0.127^†^
Using toilet		6.9 (± 0.3)		6.6 (± 0.7)	0.063^†^
Sphincter control					
Bladder management		6.4 (± 1.2)		5.3 (± 2.0)	0.003^†^
Bowel management		6.5( ± 0.9)		6.6 (± 0.9)	0.810^†^
Transfer to/from bed		6.7 (± 0.4)		5.3 (± 2.0)	0.142^†^
Wheelchair		6.8 (± 0.4)		6.6 (± 0.9)	0.024^†^
Transfer to/from toilet		6.7 (± 0.5)		6.5 (± 0.7)	0.094^†^
Mobility					
Transfer to/from bathtub or shower		6.5 (± 0.7)		6.5 (± 0.9)	0.045^†^
Walking/wheelchair		6.5 (± 0.7)		6.1 (± 1.0)	0.094^†^
Stairs		6.0 (± 0.7)		117.8 (± 7.4)	P < 0.001^†^
FIM total		117.9 (± 7.4)		117.8 (± 7.4)	0.010^†^

^a^SD = standard deviation; ^*^Chi-square test; ^1^Handgrip strength; values: from 0 to 50 kg; ^†^Student t test; ^2^Values: from 1 to 4, going from worst to best performance respectively; ªLL = lower limb; ^‡^Mann-Whitney test; ^3^Values: from 1 to 12, going from worst to best performance respectively; ^b^Short Physical Performance Battery; ^c^FIM: Functional Independence Measure; ^4^Values: from 1 to 7, going from complete dependence to complete independence, respectively.

**Figure 1.**
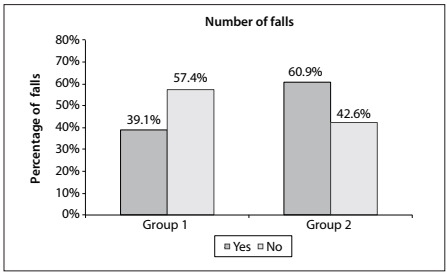
Comparison between group 1 (n = 46) and group 2 (n = 94) according to the percentage of falls.

The two resulting groups were then compared in relation to fall occurrence over the preceding year and a significant difference was found, such that Group 1 had a lower tendency to falls than shown by individuals in Group 2 (Figure 1).

## DISCUSSION

The study revealed that there was a high prevalence of falls over the preceding year among the elderly subjects (51.0%), considering that most of them fell twice or more often (56.2%). This was similar to the results obtained in the Brazilian study developed by Fabrício et al.,^1^ in which a percentage of falls of 54.0% was found among elderly people from an inpatient unit of a public hospital. However, in epidemiological investigations, the percentages of elderly that reported falls in the last year are lower, as observed in the studies of Siqueira et al.^3^ and Perracini and Ramos^7^ with values of 34.8% and 31.0% respectively. This difference is possibly due to the specific characteristics of the sample in the present study, such as high levels of frailness, associated diseases and medicines used. These characteristics were compatible with the outpatient origin of our patients and, according to the literature, they constitute fall predictors.^13^^,^^14^^,^^15^

Cluster analysis showed some characteristics that were related to falls and sociodemographic, physical, clinical and functional factors among the elderly subjects. The comparison analysis identified that the rate of occurrence of such accidents differed between Groups 1 and 2 (P = 0.042), which were shown in the analysis to present smaller and bigger tendencies, respectively. This difference shows that falls are an important factor to be evaluated as part of health professionals’ practice.

The greater expression tendency observed in Group 2, which was composed mostly by single women, is in accordance with the literature, as shown by Fabrício et al.^1^ and Moreira et al.,^16^ with regard to the rate of occurrence of falls among women, as well as by Mahoney et al.^17^ and Perracini and Ramos,^7^ who revealed that there was a significant difference between groups of elderly people that fell and did not fall, in relation to the condition of being married or not.

The numbers of medications in use and associated illnesses were not determinant in making a distinction between the groups, although the literature indicates that both are significantly related to occurrences of falls.^14^^,^^18^^,^^19^ On the other hand, such occurrences may have been due to the debilitated nature of the groups studied.

Fabrício et al.^1^ identified falls as an indicator of undiagnosed illness, and as a starting point for a more detailed evaluation. In addition, the results from the present and other similar studies demonstrate that prevention of these occurrences seems to be important, given that the consequences may be serious and in some cases can lead to death. Regarding physical and functional characteristics, predominance of low SPPB scores and high FIM scores was observed. These findings indicate that most elderly people have low physical performance concomitantly with either complete or partial independence in their daily activities, which corroborates other studies carried out in outpatient and hospital settings.^6^^,^^20^

Another point to be considered is that, despite low physical ability with regard to strength, gait and balance, the elderly are generally able to carry out daily tasks, even if slowly and with more difficulties. The way in which these tasks are carried out and their speed are not detailed in FIM, but it is pointed out in the literature that for the elderly to maintain a better quality of life, it is essential for them to do as many tasks as possible, regardless of how these tasks are done.^21^^,^^22^

The elderly people in Group 2 presented low performance relating to HS, which was considered by Fried et al.^13^ and Rolland et al.^23^ to be one of the frailness or mortality indicators among the elderly, respectively. In another study that we developed,^22^ subjects with advanced age who fell presented lower mean HS than did those who did not fall, with a significant difference between the two groups, similar to the results from the present investigation.

Shechtman et al.^24^ found significant differences relating to sex, age, functionality and motor, visual and cognitive decline, in a comparative analysis on elderly people with high and low HS. This finding indicates that low HS among elderly people who fell is related to other factors that are usually connected with falls, in accordance with the characteristics of Group 2 in this study. Regarding physical performance measured by SPPB, the analysis showed that there was low total physical performance among the individuals with a tendency to fall (Group 2), especially in relation to the balance and gait domains. Rolland et al.^23^ found that, regarding the association between physical performance and mortality among community-living elderly individuals, low SPPB scores for gait speed and HS were the parameters mostly associated with comorbidity and mortality.

Muscle weakness, sarcopenia (i.e. deterioration due to aging) and the sedentary lifestyle of most elderly people are in some studies associated with fall occurrence, imbalance and osteoporosis.^16^^,^^25^ However, in the present study, no significant difference was found between Groups 1 and 2 concerning lower-limb muscle strength. Regardless of the satisfactory reliability obtained in SPPB validation in Brazil,^26^ this can be explained by the limitation of indirect lower-limb strength in the sit-to-stand movement, in relation to repetition and time taken to perform.

There was a significant difference between the groups concerning the total FIM score, i.e. Group 1 seemed to be more independent in activities of daily living (ADLs) in comparison with Group 2. This was also observed by Saverino et al.^6^ and Sai et al.,^20^ but differed from the data of the study that did not find any difference between groups of elderly people who fell and did not fall.^27^

With regard to functionality in ADLs, the present study identified significant differences between Groups 1 and 2, concerning the motor dimension, in self-care tasks such as having a bath, dressing (upper body) and bladder control and in mobility-related tasks regarding toilet transfer and use of stairs (P < 0.001). Ruchinskas^28^ found significant differences in relation to self-care and dressing of the upper body, similarly to the present study, but did not find differences regarding the use of stairs.

In view of the limitations of this study, some recommendations deserve further attention, such as expansion of the sample size, longitudinal studies and better characterization of the falls. Furthermore, it seems important that government action is taken towards developing educative measures for fall prevention and implementation of exercises that enable safe gait and improve dynamic and static balance, with the objective of providing the elderly with greater functionality and movement stability.

## CONCLUSION

The results allow the conclusion that single female elderly people with worse physical performance and less independence in motor tasks relating to self-care (having a bath, sphincter control, toilet transfer and use of stairs) present a higher chance of belonging to the group that suffers more falls.

Identifying factors related to falls among elderly is important in helping to detect the risks of occurrences of falls, as well as for proposing strategies for preventive care and/or rehabilitation among elderly people who have already suffered falls.
